# Radar-Based Heart Sound Detection

**DOI:** 10.1038/s41598-018-29984-5

**Published:** 2018-07-26

**Authors:** Christoph Will, Kilin Shi, Sven Schellenberger, Tobias Steigleder, Fabian Michler, Jonas Fuchs, Robert Weigel, Christoph Ostgathe, Alexander Koelpin

**Affiliations:** 1 Institute for Electronics Engineering, Friedrich-Alexander-Universität Erlangen-Nürnberg (FAU), 91058 Erlangen, Germany; 2 Department of Palliative Medicine, Universitätsklinikum Erlangen, Comprehensive Cancer Center CCC Erlangen - EMN, Friedrich-Alexander-Universität Erlangen-Nürnberg (FAU), 91054 Erlangen, Germany; 3 Chair for Electronics and Sensor Systems, Brandenburg University of Technology, 03046 Cottbus, Germany

**Keywords:** Cardiology, Quality of life, Biomedical engineering, Electrical and electronic engineering

## Abstract

This paper introduces heart sound detection by radar systems, which enables touch-free and continuous monitoring of heart sounds. The proposed measurement principle entails two enhancements in modern vital sign monitoring. First, common touch-based auscultation with a phonocardiograph can be simplified by using biomedical radar systems. Second, detecting heart sounds offers a further feasibility in radar-based heartbeat monitoring. To analyse the performance of the proposed measurement principle, 9930 seconds of eleven persons-under-tests’ vital signs were acquired and stored in a database using multiple, synchronised sensors: a continuous wave radar system, a phonocardiograph (PCG), an electrocardiograph (ECG), and a temperature-based respiration sensor. A hidden semi-Markov model is utilised to detect the heart sounds in the phonocardiograph and radar data and additionally, an advanced template matching (ATM) algorithm is used for state-of-the-art radar-based heartbeat detection. The feasibility of the proposed measurement principle is shown by a morphology analysis between the data acquired by radar and PCG for the dominant heart sounds S1 and S2: The correlation is 82.97 ± 11.15% for 5274 used occurrences of S1 and 80.72 ± 12.16% for 5277 used occurrences of S2. The performance of the proposed detection method is evaluated by comparing the F-scores for radar and PCG-based heart sound detection with ECG as reference: Achieving an *F*1 value of 92.22 ± 2.07%, the radar system approximates the score of 94.15 ± 1.61% for the PCG. The accuracy regarding the detection timing of heartbeat occurrences is analysed by means of the root-mean-square error: In comparison to the ATM algorithm (144.9 ms) and the PCG-based variant (59.4 ms), the proposed method has the lowest error value (44.2 ms). Based on these results, utilising the detected heart sounds considerably improves radar-based heartbeat monitoring, while the achieved performance is also competitive to phonocardiography.

## Introduction

Continuous heart rate monitoring and instantaneous heartbeat detection are key parameters in modern vital sign monitoring. Current methods require touch-based wiring of the patient’s skin, e.g., electrocardiography as gold standard method^1^. Therefore, an intensive monitoring is solely feasible in the setting of an intensive or intermediate care unit, but beneficial effects are to be expected in regular hospital wards, as well as in an out-patient setting. Continuous monitoring of heartbeat and respiration can lead to a better outcome by early detection and prevention of critical states of health and additionally contributes to their prevention^2^. Various common clinical conditions are immediately hazardous and potentially fatal, e.g., cardiac arrhythmia^3^, heart attacks^4^, and stroke^5^, sudden death in infants^6^, and epilepsy^7^, or may lead to deterioration of health, e.g., hypertension and arteriosclerosis^8^. Continuous monitoring can improve outcome for persons at risk and facilitate early detection. Thus, an alternative is necessary, which is reliable, convenient by enabling monitoring of fully clothed persons or through blankets for patients lying in beds, and feasible in every day conditions.

A more comfortable way to monitor a person’s vital signs is enabled by ballistocardiography^9,10^, but applicability is restricted to bed-ridden subjects and standardised conditions. An approach for long term vital sign monitoring of a single person-under-test (PUT) are wearable systems. Next to complex sensor systems^11^ and infrared camera-based systems, which require several markers on the chest of the PUT^12^, a variety of miniaturised sensors have been published. These tiny pressure^13,14^, motion^15^, mechano-acoustic^16^, and photoplethysmography^17^ sensors can easily be integrated in modern wearables, e.g., smartwatches. In contrast to the electrocardiograph (ECG), these methods do not utilise the electrical effect of the heartbeat but the cardiomechanical effects^18–20^. Muscular action of the heart leads to a pulsatile increase and decrease of pressure in the arteries. Depending on their elasticity, the arteries’ local volume will change, as pressure changes. Change of local pressure and volume can be measured over time and correlates with the heartbeat^18,19,21^. Additionally, contraction of the heart and consecutive closure of the heart’s valves lead to a vibration, which is transmitted through the tissues of the chest and can be heard on the surface, e.g., by placing a stethoscope on the skin^19,22^. This instrumentation is called phonocardiograph (PCG) and is used for heart sound analysis^23,24^. Regarding clinical issues, a PCG can be utilised to assess dysfunctions of cardiac valves^25^, as well as to monitor cardiac performance during anaesthesia^26^.

Nevertheless, the most comfortable approach is touch-free measurement of the vibrations on the human body caused by the contraction of the heart muscle. This is enabled by distance, displacement or vibration measuring sensor concepts like laser-based^27–32^ or radar-based^33–41^ measurement systems. While laser sensors have a very high distance resolution and can be accurately focused on specific measurement spots, using radar systems offers convenient heartbeat detection, since the emitted electromagnetic wave penetrates clothing and bedding. Additionally, utilising beamforming and modulation techniques, multiple persons can be simultaneously monitored by one radar system, as well as multiple measurement spots on one PUT^42^. Especially radar-based sensor systems can be easily integrated within the environment of the PUT, e.g., distributed at home to enable further measurement possibilities regarding continuous vital sign monitoring. Almost all previously published laser and radar-based measurement systems extract the low frequency pulse wave component, whose main frequency corresponds to the heart rate of the PUT, and analyse the signal’s spectrum by utilising the fast Fourier transform (FFT)^27–29,33–38,41^. Since the FFT requires a comparatively long observation time to accurately detect the heart rate, algorithms, which provide instantaneous information on heartbeats will be a vast improvement^43,44^. Furthermore, by detecting beat-to-beat intervals, heart rate variability (HRV) can be assessed, which is the change of heart rate over the time^45^. HRV is an uprising research topic and has multiple clinical implications, e.g., diagnosis of depression and burn-out, as well as epileptic seizures and risk of sudden cardiac death^30,40^. While various ballistocardiography-based^46,47^, as well as laser-based^30–32^ heart sound measurements were published, only Mikelshon *et al*.^39^ and Aardal *et al*.^48^ reported higher frequency components by radar, which were neither assigned to a specific source nor individually segmented and analysed. An interbeat interval (IBI) determination by heart sound classification promises more accurate results, since the published pulse component-based heartbeat curves differ in shape, as well as timing regarding the distances between heartbeat and ECG signal peaks^36,37,43,44^.

In this paper, radar-based heart sound detection is presented for the first time. A logistic regression hidden semi-Markov model (HSMM)-based heart sound segmentation, similar to^49^, is proposed, which enables further cardiac diagnostics by analysing the classified heart sounds^23,24^ and the HRV^45^. Tracing back the higher frequency components in the radar signal to their physiological source and proving the feasibility of radar-based heart sound detection is the key aspect in this paper, in which an unmodulated continuous wave radar system in an interferometric configuration for relative displacement measurements was used. In addition to the radar signal, data from medical PCG, three channel ECG and respiration sensor (RS) for validating experimental data were synchronously captured. The vital signals of eleven persons under various measurement conditions, e.g., while breathing at leisure, during breath-holding, and after physical exertion, were monitored and 9930 seconds of measurement data were collected in total. Correlation with physiological data from cardiac action is provided by standardised assessment. Validation of the feasibility of radar-based heart sound detection and the algorithm’s functionality is provided by simultaneously evaluating the ECG and PCG data. Additionally, a correlation-based morphology analysis between radar and PCG data is presented. Furthermore, the respiratory influence on the heart sounds is investigated by means of the peak envelope variation, as well as the split of the second heart sound, two specific research topics in PCG-based cardiovascular physiology. The performance of the proposed algorithm is evaluated by comparison with ECG and PCG measurements, as well as pulse wave detection-based algorithms. The novelty of the presented results are substantiated in a discussion and, finally, the insights of the utilised methods are presented.

## Results

### Heart sound validation

**Figure 1:**
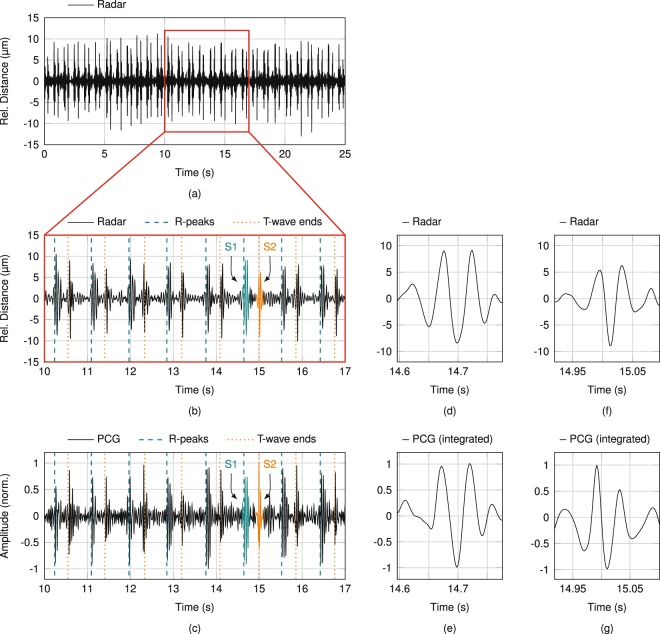
(**a**) Exemplary filtered radar signal with the xiphoid process in focus. (**b**) Enlarged cutout with two highlighted heart sounds S1 and S2. (**c**) Synchronised PCG measurement data with corresponding heart sounds highlighted. (**d**) Enlarged versions of the highlighted S1 in radar and (**e**) PCG data. (**f**) Enlarged versions of the highlighted S2 in radar and (**g**) PCG data.

The feasibility of radar-based heart sound detection is investigated by comparison with ECG data as gold standard method for heartbeat detection and with PCG data as gold standard method for heart sound detection, in particular. Figure 1 shows the sensor signals for an exemplary measurement, in which the PUT was stationary with resting heart rate (60…80 bpm) and breathing at leisure (8…12 breaths per minute). The radar antenna was focused on the xiphoid process, while the PCG was positioned at the fourth intercostal space at the left side (4 L). Both ROIs are illustrated in Fig. 8(a). A 25-second window of the acquired radar distance data is shown in Fig. 1(a). Frequent occurrences of two subsequent, short vibrations with an amplitude of approximately 10 m are clearly visible. A cutout of 7 s is plotted enlarged in Fig. 1(b), in which correlation with the detected R-peaks and T-wave ends in the ECG signal can be observed. The hypothesis of this paper is that theses vibrations correspond to the heart sounds, which is proven in the following. In accordance to cardiovascular physiology, the first vibration (S1) immediately follows the R-peaks, while the second vibration (S2) occurs near the T-wave ends. The corresponding heart sound cutout of the synchronised PCG data is shown in Fig. 1(c). Both cutouts visually show high similarities regarding signal shapes, as well as time of occurrences of the detected vibrations. While the timing of occurrences is analysed in the following subsection, this paragraph deals with a morphology analysis of both heart sounds. The importance of a morphology analysis is discussed in Papadaniil *et al*.^50^. The highlighted heart sounds in Fig. 1(b and c) are depicted enlarged in Fig. 1(d–g). The morphological comparison is conducted by correlation. Since PCG are based on acceleration, whereas radar data represents the distance, one of the data has to be converted to fit the same domain for both sensing concepts for correlation. In principle, the radar data can be differentiated twice, but this will lead to an overweighting of the noise component artificially lowering the correlation result. Therefore, the PCG data is integrated twice to fit the data representation of the radar. The Pearson correlation coefficient is 91.41% for the shown S1 signals of radar and PCG, respectively, in Fig. 1(d and e) and 92.35% for the shown S2 signals in Fig. 1(f and g). The overall correlation of the heart sounds in Fig. 1 is 91.54 ± 3.19% for S1 and 80.98 ± 11.53% for S2. Evaluating the complete database by utilising all heart sounds with sufficient signal-to-noise ratio (SNR) results in similarly high values: The correlation between radar and PCG data is 82.97 ± 11.15% for 5274 used occurrences of S1 and 80.72 ± 12.16% for 5277 used occurrences of S2. The differences to a perfect correlation of 100% can be addressed to different sources: For the radar, the antenna characteristic has some influence as well as the integrating behaviour of the relatively large ROI compared to the PCG^44^. The morphology also varies in PCG-based measurements, since the pressure, which is used to push the PCG on the ROI, highly influence the measurement signal. Furthermore, to minimise any mutual influence between PCG and radar sensor due to micro-vibrations of the manually positioned PCG, the corresponding ROIs were chosen slightly different with no overlap, but close to each other for the radar and the PCG during the simultaneous measurements, which also results in different signal morphologies and is hard to compensate for. Also the sizes of both ROIs are different. Nevertheless, the measured radar-based heart sound signals show high correlation regarding timing and signal shape to the PCG signals.

### Timing analysis

**Figure 2:**
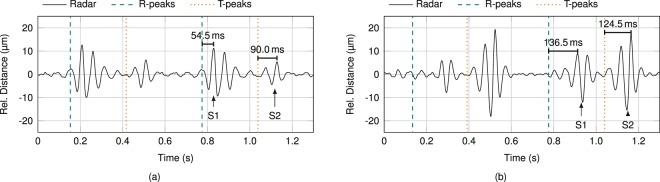
Timing analysis of S1 and S2 at (**a**) 4 L and (**b**) CL.

PCG and radar data in Fig. 1(a) representing heart sounds show a high similarity provided both sensors are placed in close proximity. As phonocardiography constitutes touch-based measurement of high frequency vibrations on the surface of the body, PCG and radar detect the same physiological phenomenon, but in different domains (acceleration versus distance). In case of detection of heart sounds, surface vibrations are caused by mechanical action of the heart. To investigate the propagation velocity of these vibrations, signal timings at different ROIs have to be evaluated, which are depicted in Fig. 8(a). In Fig. 2 the exact timing of S1 and S2 occurrences in the radar signal is analysed for two ROIs in the same PUT. While the antenna was focused on 4 L in Fig. 2(a), CL was focused on in Fig. 2(b). Next to the radar signal, significant characteristics of the ECG signal are marked, as well. Since the T-peak represents a more explicit reference point than the T-wave end, the T-peaks are shown together with the R-peaks in these plots. Comparing the included timing labels, two findings are observed. First, intervals between R-peak and S1, as well as T-peak and S2 are larger in CL comparison to 4 L. Second, increase of interval is heightened between R-peak and S1 in comparison to T-peak and S2. This phenomenon is accounted for by the different points of origin of S1 and S2 in reference to the ROIs. ROI in CL is approximately 0.30 m further remote from S1 than ROI in 4 L, whereas only 0.15 m from S2. Based on these approximations for an average PUT, propagation velocities of 3.8 $\frac{{\rm{m}}}{{\rm{s}}}$ and 4.0 $\frac{{\rm{m}}}{{\rm{s}}}$, respectively are calculated from the radar data. While the measured propagation velocities vary between PUT, they are approximately equal for each single PUT. This implies that both heart sounds propagate in the same physical manner. The propagation velocity range for all PUT is about 4…9 $\frac{{\rm{m}}}{{\rm{s}}}$, which corresponds to the values published in literature^51–53^. In addition, this value range corresponds to investigated pulse wave velocities, which implies transversal dispersion through tissue and along anatomic surfaces as equal kind of propagation^51^.

### Respiratory influence

In this section, the respiratory influence on the heart sounds is analysed utilising the database. The heart sound signal extracted from the radar data is compared with the synchronous RS signal confirming two physiological aspects, peak envelope variation of heart sounds, as well as S2 split during inspiration.

#### Peak envelope variation

**Figure 3:**
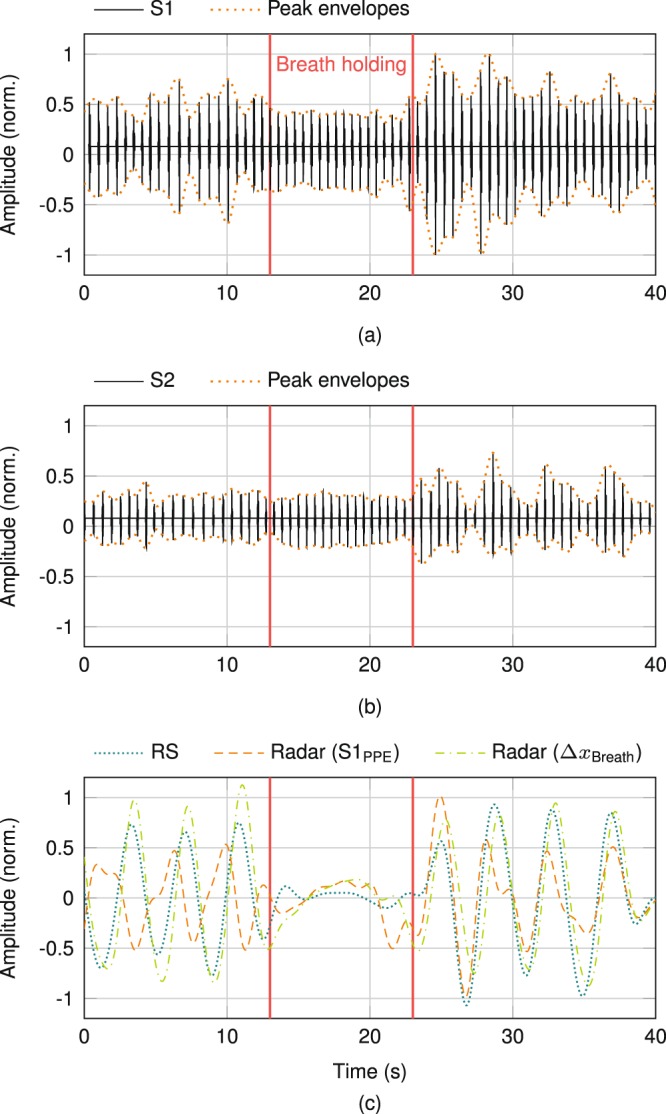
(**a**) Peak envelope variation of S1 and (**b**) S2. (**c**) Breathing influence on the S1 peak envelopes by comparing its PPE to the RS, as well as to the extracted breathing data from the radar signal.

To investigate the respiratory induced peak envelope variation, S1 and S2 each have to be extracted individually from the heart sound signal. Here, an HSMM-based heart sound segmentation^49^ was optimised for radar measurements and used to segment the heart sound signal in four distinct partitions: S1, systole (without S1), S2, and diastole (without S2). The resulting analysis is illustrated in Fig. 3. While Fig. 3(a) only contains the extracted S1 segments, the S2 segments are shown in Fig. 3(b). Additionally, the upper and lower peak envelopes are depicted in both sub-plots. The peak-peak envelope (PPE) of each heart sound (S1_PPE_ and S2_PPE_) is defined as the difference between upper and lower peak envelope. The distance-based breathing part of the detected distance variation (Δ*x*_Breath_) due to thorax movements was extracted from the radar signal by a fourth order Butterworth filter with a passband of 0.1…0.5 Hz. The RS signal and S1_PPE_ were filtered likewise. A comparison between these filtered signals, the RS signal, the S1_PPE_, and the radar distance-based breathing signal Δ*x*_Breath_ is shown in Fig. 3(c). The PUT was breathing normally for the first 13 s, followed by 10 s of breath-holding, and breathing at leisure for the remaining time under observation. Breath-holding is observable in all signals of Fig. 3(c), since amplitude changes decrease considerably with breath-holding. Furthermore, the radar distance-based respiratory signal is highly similar to the signal from the RS, while PUT breathes at leisure: The correlation between both respiration signals is 94.44% for the presented measurement data and 89.14 ± 10.53% for the complete database. The S1_PPE_, in contrast, shows a contradictory behaviour. While after breath-holding this signal equals both other signals, too, the curve is reversed before breath-holding. In general, the heart sound PPE, both for S1 and S2, are linearly dependent on breathing, either positively or invertedly. Evaluating the database, the sign of each PPE curve discloses a random behaviour, since comparing measurements does not show any regularity. While in some measurements the PPE curves of both heart sounds are equal, they are opposed in other ones. Neither can any regularity be found for an individual PUT, nor for a specific ROI. Reversal of PPE after breath-holding was observed in each PUT, but not consistently. These peculiar observations regarding the heart sound PPE are not caused by erroneous radar data, but confirm a known phenomenon in literature^54–56^.

#### S2 split

**Figure 4:**
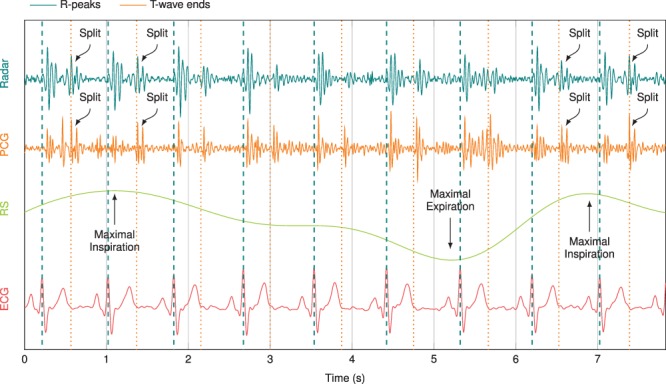
A split of S2 in its aortic and pulmonary parts during inspiration can be observed both in the PCG signal measured at 2 L and in the radar signal measured at 2 R. The timing of the heart sounds is verified by the R-peaks and T-wave ends of the ECG signal.

Next to the peak envelope variation, a split of the second heart sound is occasionally measured, which is illustrated in Fig. 4. This non-pathological S2 split only occurs during inspiration and, according to literature^57,58^, is observed mostly for younger persons. Here, S2 is split into its two components, the aortic (A2) and pulmonary (P2) parts. While the stroke volume of the right ventricle increases during inspiration, the stroke volume of the left ventricle decreases, which leads to an extended systole of the right heart (pulmonary circulation) and a shortened systole of the left heart (body circulation), respectively. Hence, the aortic valve closes earlier and the pulmonary valve later, which results in a dispersed second heart sound. In Fig. 4, the physiological timing of radar-based data on heart sounds is substantiated by the R-peaks and end of T-waves of the ECG signal, while the RS signal after application of the bandpass filter with a passband of 0.1…0.5 Hz is used as reference. The S2 split is visible in the PCG signal measured at the second intercostal space at the left side (2 L), as well as in the heart sound signal extracted from the radar data measured at the second intercostal space at the right side (2 R). Both ROIs are illustrated in Fig. 8(a). While the gap between A2 and P2 is normally around 35.0 ms in the PCG data, the gap increases to approximately 62.5 ms during inspiration, which conforms to^57^. For the radar data, the gap increases from approximately 38.5 ms to about 66.0 ms. The slightly higher values are based on the lateral integrating behaviour of the radar system over a larger measurement spot^44^.

### Performance analysis

Performance of the HSMM-based heart sound segmentation is analysed regarding two criteria. First, the usability of radar systems is compared to PCG evaluating the correctness of the heart sound segmentation by means of the F-score (*F*_1_). Second, the timing accuracy of this novel radar-based heartbeat detection method derived from heart sounds is compared to a pulse wave evaluating algorithm as state-of-the-art method regarding the obtained IBI values.

#### F-score

The F-score is a common measure for test accuracy, which considers both the precision *p* and the recall *r*^59^:

1 $${F}_{{\rm{1}}}=2\cdot \frac{p\cdot r}{p+r}\mathrm{.}$$

A cross-validation is applied on the complete measurement database with 9930 seconds of synchronised sensor data to calculate the F-scores, which each consist of a mean value and the standard deviation. While the data of six PUT are used to train the model, subsequently, residual data of five PUT are used for testing. All 462 possible combinations were evaluated to calculate the F-scores for PCG, as well as radar-based heart sound detection. In Table 1, the F-scores are compared for two HSMM variants, the model presented by Springer *et al*.^49^ (variant A) and the adjusted variant proposed in this paper (variant B). The F-scores for each individual heart sound are presented (*F*_1,S1_ and *F*_1,S2_), as well as the joint F-scores (*F*_1,AVG_). The upper lines in both variants show the training results in italic, while the test results are shown in the lines below. The adjustments of the algorithm parameters in variant B slightly decrease the performance of the PCG, but strongly increase the performance of the radar system. Since the F-scores for radar systems with adjusted parameters almost equal the values of the gold standard method, PCG with variant A, the feasibility of radar-based heart sound detection is confirmed. The lower mean values of the radar system F-scores, as well as the higher standard deviations are based on the radar’s lower heart sound SNR values. By Schmidt *et al*.^60^, these SNR values were defined as the ratio of the heart sound segment amplitudes and the amplitudes of the subsequent systole segment and diastole segment, respectively. The lower SNR values of 7.11 dB for S1 and 3.29 dB for S2, in comparison to 7.81 dB (S1) and 5.69 dB (S2) for the PCG data, are due to the limited dynamic range of the used radar system.

**Table 1:** Comparison of the F-scores in training (italic values) and test (standard typeface) between PCG and radar-based heart sound detection for two algorithm variants.

Variant^1^	BP filter (Hz)^2^	ACF limits (s)^3^	PCG			Radar		
			*F*1,S1 (%)	*F*1,S2 (%)	*F*1,AVG (%)	*F*1,S1 (%)	*F*1,S2 (%)	*F*1,AVG (%)
A	25…400	0.50…2.00	*94.46* ± *1.41*	*94.61* ± *1.37*	*94.53* ± *1.38*	*87.10* ± *2.89*	*86.13* ± *3.09*	*86.61* ± *2.97*
			*94.09* ± *1.64*	*94.22* ± *1.59*	*94.15* ± *1.61*	*86.27* ± *3.37*	*85.37* ± *3.53*	*85.82* ± *3.43*
B	16…80	0.45…1.45	*93.56* ± *1.52*	*93.03* ± *1.54*	*93.30* ± *1.52*	*93.85* ± *1.48*	*91.74* ± *1.85*	*92.80* ± *1.62*
			93.16 ± *1.80*	*92.60* ± *1.97*	*92.88* ± *1.88*	*93.33* ± *1.91*	*91.11* ± *2.31*	*92.22* ± *2.07*

^1^A: HSMM model presented by Springer *et al*.^49^, B: proposed HSMM model with adjusted settings^2,3^.

^2^Passband range of the applied bandpass (BP) filter.

^3^Heart cycle limits for the utilised autocorrelation (ACF)-based heart rate estimation.

#### Root-mean-square error

The root-mean-square error (RMSE) of the IBI values is a further performance analysis measure, which represents the timing accuracy of detecting single heartbeats. In general, the RMSE describes the deviation of a measurand from a reference value. Before calculating the RMSE, all individual heartbeats have to be detected in the ECG signal as gold standard reference, as well as in the comparing measurement signals. The R-peaks in the ECG signal are defined as reference instants of time of heartbeat occurrence, and therefore, the distance between two R-peaks is used for IBI calculation. Regarding heart sound-based heartbeat detection, the starts of the S1-segments are used for the PCG data, as well as the radar data. The radar data are additionally bandpass filtered in a range of 0.75…3.0 Hz to obtain the pulse wave component, which is utilised by a state-of-the-art heartbeat detection algorithm for radar systems^43^. This advanced template matching (ATM) algorithm uses the detected pulse wave peaks to determine single heartbeat occurrences. For all measurement signals, the final IBI values are calculated once per second by averaging the five latest determined heartbeat intervals with a median filter. These IBI values are used to determine the RMSE:

2 $${\rm{RMSE}}=\sqrt{\frac{{\sum }_{t=1}^{n}{({I}_{t,{\rm{ECG}}}-{I}_{t})}^{2}}{n}}\mathrm{.}$$

**Figure 5:**
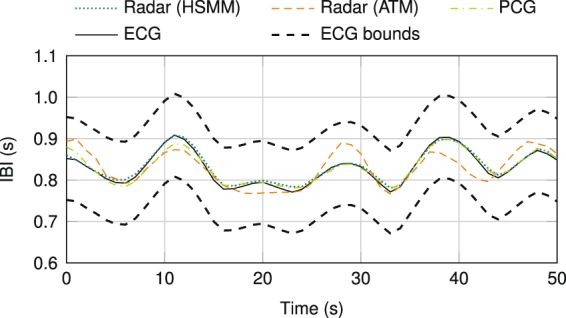
IBI curve comparison of a PCG measurement and two algorithms evaluating a radar measurement to ECG data as reference.

Here, *I*_*t*,ECG_ is the IBI value obtained from the ECG signal as reference at time instance *t*, *I*_*t*_ is the simultaneously determined IBI value of the system to be compared to the reference, and *n* represents the number of time instances. An exemplary IBI curve comparison between all measurement systems is plotted in Fig. 5. Both curves belonging to heart sound-based heartbeat detection systems, radar (HSMM) and PCG, almost equal the IBI curve of the ECG as reference. While the IBI curve of the pulse wave detection-based variant, radar (ATM), is permanently within the ECG bounds of ±0.1 s for the complete time window of 50 s, its deviation from the ECG curve is considerably larger. In contrast to the mean RMSE value of 144.9 ms for the ATM variant, considering all IBI values of the complete database, the RMSE of 44.2 ms for radar-based heart sound detection is considerably smaller. The RMSE for PCG-based heart sound detection is larger with a value of 59.4 ms, despite the PCG’s higher F-scores. While the deviation of *I*_*t*_ from *I*_*t*,ECG_ is maximally 400 ms for radar measurements, few outliers for PCG measurements with values up to 1000 ms result in a worse RMSE. Here, the individually superior HSMM variant was chosen to calculate the RMSE values, variant A for the PCG data and variant B for the radar data, respectively.

## Discussion

In this paper, the feasibility of radar-based heart sound detection was investigated. While electrocardiac activities have been always measured by the gold standard device ECG, measuring the diversity of physiological implications demands various methods. The emitted pulse wave due to heart’s contraction can be measured by a pressure capsule or photoplethysmography, at which the signal shape is highly dependent on the ROI. The heart sounds, caused by muscle contraction and valve closures, are detected by phonocardiography. Since heart sounds only negligibly transit the body as an acoustic wave, but rather propagate as a mechanical wave along arteries and vessel walls^51–53^, the PCG measures the consequential vibrations of the body surface due to nearby vessel modulations. As these modulations and the pulse wave effects on the body surface^44^ are of similar type, distance measuring devices like laser or radar systems simultaneously detect both cardiophysiological effects.

The implication that radar systems detect heart sounds in addition to the pulse wave component was investigated by a multiplicity of measurements. Exemplary validation measurements show high similarities between radar and PCG data regarding heart sounds’ occurrence timings and signal shapes. Furthermore, the detected heart sounds by radar have a high conformity concerning cardiophysiological correlation to the ECG signal. In addition to Springer *et al*.^49^, the ROIs, as well as diverse standardised conditions, e.g., breath-holding and physical exertion, were varied for all PUT. The presented morphology analysis between radar and PCG data proves the feasibility of radar-based heart sound detection. A correlation of 82.97 ± 11.15% for S1 and 80.72 ± 12.16% for S2 are excellent values, considering the differences regarding measurement principles, sizes of the measurement spots, and ROIs. The feasibility of the proposed detection method is substantiated by high F-scores. Applying the algorithm with the same parameters results in an *F*_1,AVG_ score of 94.15 ± 1.61% in the cross-validation test for the PCG data and 85.82 ± 3.43% for the radar data. Adjusting the algorithm parameters for radar-based heart sound detection (variant B) increases the *F*_1,AVG_ score for the radar data to 92.22 ± 2.07%, which approximates the value of PCG-based heart sound detection. The still slightly smaller F-score is caused by the lower SNR of the detected heart sounds, which can be explained by the limited dynamic range of the utilised radar system, and which is substantiated by the higher variations of the F-scores. Also specific heart sound characteristics, like respiration induced peak envelope variation, as well as the S2 split during inspiration, are detectable with radar systems. For the first time, radar technology is proven to be able to monitor heart sounds. The exemplary validation measurements, the high correlation values between radar and PCG data, and the similar *F*_1_ values affirm radar systems as an alternative method to phonocardiography. With radar systems, diagnostics is feasible from a distance of several meters and in everyday settings, e.g., penetrating clothing or bedding. Additionally, the theoretical considerations regarding the heart sound propagation were validated by specific measurements for timing analyses.

Furthermore, utilising the detected heart sounds considerably improves radar-based heartbeat detection, an emerging research topic in continuous vital sign monitoring. This has been shown by comparing the averaged errors of the detected IBI values for the proposed measurement principle to a state-of-the-art radar-based heartbeat detection algorithm. While the RMSE for the ATM algorithm^43^ has a value of 144.9 ms, the proposed HSMM utilising heart sound segmentation-based algorithm considerably reduces the RMSE to 44.2 ms. Hence, detecting heart sounds contributes considerably to enhance radar-based heartbeat detection. Last but not least, radar systems simultaneously monitor two cardiophysiological components, i.e. heart sounds, as well as the pulse wave, combining multiple sensors in one touch-free measurement system. Furthermore, the velocity of the pulse wave can be estimated from the radar measurements.

## Methods

### Six-Port interferometry

For the generation of the radar signals offering the extraction of the heart sounds, a Six-Port interferometer has been used as alternative distance sensing concept. The Six-Port architecture was introduced by Engen and Hoer in the 1970s for power measurements^61^. Nowadays, this architecture is also utilised as a quadrature interferometer for microwave radar applications^62^. The complete passive receiver structure consists of one Wilkinson power divider and three quadrature hybrid couplers, which implicates a high phase accuracy along with a low power consumption and low costs. The Six-Port network is fed by two input signals at ports *B*_1_ and *B*_2_ that are superimposed within the Six-Port network with static relative phase shifts of *n* ⋅ *π*/2 (*n* ∈ {1, 2, 3, 4}) amongst each other, resulting in four output signals *B*_3...6_. Since the relative phase shifts between the four output signals are multiples of *π*/2, the baseband (BB) signals can be transformed into a complex representation $\underline{Z}$. The in-phase, as well as the quadrature component are two differential pairs, which are orthogonal to each other:

3 $$\underline{Z}=I+jQ=({B}_{5}-{B}_{6})+j({B}_{3}-{B}_{4}\mathrm{).}$$

The ideal complex representation of a linear phase shift is a movement along the unit circle. Therefore, the argument of the complex expression $\underline{Z}$ represents the relative phase shift Δ*σ* between the two input ports:

4 $${\rm{\Delta }}\sigma ={\rm{\arg }}\{\underline{Z}\}={\rm{\arg }}\{({B}_{5}-{B}_{6})+j({B}_{3}-{B}_{4})\}\mathrm{.}$$

The four output signals of the Six-Port structure *B*_3...6_ code this Δ*σ* by proportional power. Therefore, Δ*σ* is analysed by diode power detectors converting the high frequency to close to DC in BB signal domain.

### Experimental setup

**Figure 6:**
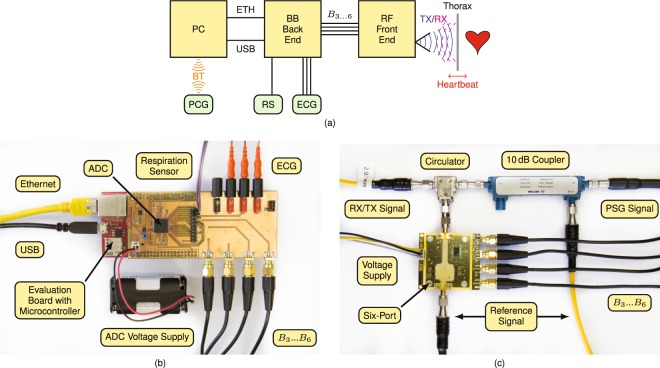
(**a**) Block diagram of the overall experimental setup. (**c**) Photographs of the BB back end and (**d**) the RF front end.

The experimental setup consists of a radio frequency (RF) front end, a BB back end with several sensors attached handling any signal close to DC or with low frequency, and a personal computer (PC) for data and signal processing. Additionally, the PCG *Electronic Stethoscope Model 3200* from *3M Littmann* is connected via Bluetooth (BT) to the PC for vital sign database generation. Figure 6(a) depicts a block diagram of the overall measurement setup. The PC is working as master and communicates with the BB back end via Ethernet (ETH), while it provides voltage supply via USB. A temperature-based airflow sensor was utilised as RS for vital sign database generation.

The BB back end consists of the microcontroller evaluation board *XMC4500 Relax Kit* by *Infineon* and an analogue-to-digital converter (ADC) feeding piggyback board. A photograph of the complete back end is shown in Fig. 6(b). While the evaluation board is supplied by USB, the piggyback board uses four external batteries to galvanically isolate the attached sensors. Next to the four BB voltages from the RF front end, three ECG electrodes and a RS are attached and sampled by the ADC. The *ADS1298* from *Texas Instruments* with eight synchronous differential channels, a resolution of 24 bit and a sample rate of 2000 samples per second is used for data acquisition. The ECG electrodes are attached at both arms of the PUT, as well as at the left leg. Since each two electrodes are sampled differentially, two leads of Eindhoven’s triangle are acquired, while the third lead can be calculated.

A photograph of the RF front end is shown in Fig. 6(c), whose main part is a Six-Port microwave interferometer, a specific type of CW radar. Since the antenna spot on the target, as well as the scattering of the reflected wave are required to be very small for an accurate heart sound detection, a monostatic system variant was built. A *PSG Analog Signal Generator E8257D* from *Keysight* is used to generate an RF signal with a frequency of 24.17 GHz. A 10 dB coupler splits the PSG signal into two parts, whereupon the major part is transmitted through a circulator to be emitted by a small horn antenna towards the PUT. The equivalent isotropically radiated power (EIRP) was measured using a *PSA Series Spectrum Analyzer E4446A* from *Keysight* and is with a value of approximately 18 dBm below the specified 20 dBm limit of the corresponding industrial, scientific and medical (ISM) radio band. The transmitting (TX) antenna is focused on the thorax of the PUT and concurrently works as a receiving (RX) antenna for the signal portions scattered back from the PUT. The received signal is fed by the circulator to one of the two Six-Port inputs. The other input port is fed by the reference signal, the coupled transmit signal of the coupler. The Six-Port output signals are down-converted to BB voltages by four Schottky diode-based envelope detectors *ADL6010* from *Analog Devices* and are fed via SMA cables to the BB back end.

### Radar data pre-processing

With *λ* = *c*/*f* being the wavelength of the carrier frequency, relative distance changes of the target can be calculated by inserting Δ*σ* in

5 $${\rm{\Delta }}x=\frac{{\rm{\Delta }}\sigma }{2\pi }\cdot \frac{\lambda }{2}\mathrm{.}$$

**Figure 7:**
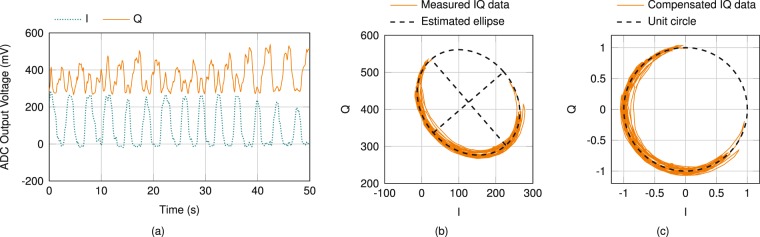
(**a**) Exemplary in-phase (I) and quadrature (Q) signals for a time frame of 50 s and (**b**) the corresponding projection in the complex plane along with an estimated ellipse, which represents BB errors induced by impairments at the RF part of the system. (**c**) Error compensated complex representation and the unit circle as ideal projection of a moving target.

Impairments at the RF front end however, like nonidealities of components, as well as reflections due to mismatches, result in amplitude and phase imbalances within the Six-Port network leading to distorted voltage amplitudes of the BB signals. The sampled BB signals are induced by offset, gain and phase errors, which deform the unit circle as ideal projection of a moving target in the complex plane to an offset ellipse. An arctangent demodulation of the uncompensated complex representation results in distorted and erroneous values for Δ*σ* and thus in faulty measured distance values. Hence, an ellipse fitting algorithm similar to Singh *et al*.^63^ has to be applied as a pre-processing step to compensate for these BB errors. In Fig. 7(a) exemplary error induced in-phase and quadrature signals are depicted, while the resulting complex representation is shown in Fig. 7(b) along with the estimated ellipse including its two defining axes. In the first step of the compensation procedure, the ellipse’s offset is subtracted from the measurement data. Afterwards, the data are rotated by the angle of the ellipse’s main axes, and lastly, the data are stretched or compressed to eliminate gain errors. Figure 7(c) illustrates the compensated complex representation of Fig. 7(b) together with the dotted ideal unit circle. Since five coarsely distributed measurement points are sufficient to estimate the ellipse, this compensation algorithm can be re-applied even during measurement operation if an changing scenario leads to modified BB errors. A successive arctangent demodulation of the compensated representation results in phase values in the range of [−*π*, +*π*), which represents a target movement range of half the wavelength *λ*. As a phase value of +*π* equals −*π*, phase jumps occur for movements outside the initial unambiguousness range. These are unwrapped by adding or subtracting 2*π* at phase jumps larger than −*π* or +*π*, respectively.

### Heart sound detection

**Figure 8:**
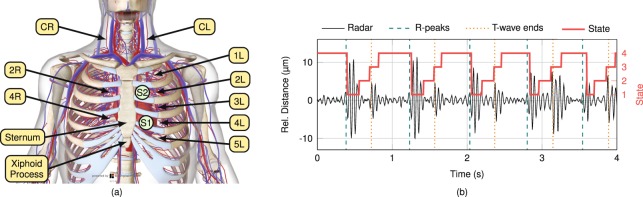
(**a**) Cardiovascular system at thorax and neck^68^. (**b**) Resulting states after HSMM-based segmentation of the heart sound signal extracted from the radar data.

Muscle cells initiate contraction upon change of the cell membrane’s electrical charge. As a result of spreading depolarisation and repolarisation the heart becomes an electric dipole, which is recorded by ECG as gold standard method to record heart action in cardiac diagnostics. The ECG signal depicts a heartbeat in form of a specific figure with a preceding smaller positive wave (P-wave), followed by a bi- or triphasic complex (QRS-complex) and ending in a higher positive wave (T-wave)^19^. Concerted heart muscle contraction following depolarisation leads to ejection of blood, whereby a mechanical pulse wave travels on the vascular walls^19,44,64^. Simultaneously, the closure of the tricuspid and mitral valves along with the subsequent isovolumetric contraction lead to a short and high frequency vibration. This is called the first heart sound (S1), which occurs directly after the R-peak in the ECG signal and can be measured by PCG. Closures of aortic and pulmonary valves at the end of ejection phase cause the second heart sound (S2), which is synchronous with the end of the T-wave. Specific areas of the human chest are optimal for detecting heart sounds, i.e. cardiac apex left of the sternum and second paramedian intercostal left space for S1 and S2, respectively. Along the greater vessels originating from the aorta, e.g., the left (CL) and right (CR) common carotid artery, heart sounds can be detected, too^19,65^. Figure 8(a) shows the cardiovascular system of torso and neck along with various ROIs for heart sound detection. The intercostal spaces on each side of the thorax are labelled by their order followed by an L (left) or R (right) for the corresponding side.

The heart sounds are extracted from radar and PCG data by applying a fourth order Butterworth filter with a passband of 16…80 Hz, as heart sounds are expected within this range^58^. The logistic regression HSMM-based segmentation of heart sounds by Springer *et al*.^49^ was adjusted and afterwards utilised to extract S1 and S2 from the filtered signals. Here, an HSMM is equivalent to a duration dependent hidden Markov model, which implies that the probability of a state change is dependent from the duration how long the system is already residing in this state. The longer the system is remaining in a state, the higher the probability of leaving this state. In general, the durations of the single HSMM states are modelled by normal distributions consisting of mean values and standard deviations. These values are empirically predefined for the first and second heart sound and represent 122 ms ± 22 ms for S1 and 92 ms ± 22 ms for S2 in healthy adults^60^. Furthermore, the system model consists of four states, which can only change in the following order: S1, systole, S2, diastole. Here, systole is defined as the complete systole without S1, diastole respectively without S2. S1 corresponds to state 1, diastole to state 4.

While HSMM-based heart sound detection for PCG^49^ is termed variant A, the proposed algorithm for radar systems is termed variant B. A major improvement of variant B is the adjusted passband range of 16…80 Hz instead of 25…400 Hz, since the signal of the physiological heart sound is below 80 Hz with mostly noise above this frequency. Integrating behaviour over a large area of the body results in additional lower frequency components as the frequency spectrum is widened. Radar-based measurements surpass PCG performance as they decrease the lower passband boundary. Furthermore, the limits of heart cycles for autocorrelation-based heart rate estimation were adjusted. The former limits of 0.5…2.0 s representing a heart rate range of 30…120 bpm are changed to 0.45…1.45 s, which favours heart rates of 41…133 bpm. While heart rates below 40 bpm are uncommon for healthy adult PUT, heart rates above 120 bpm can occur after physical activity. Two-sided *t*-tests on 462 samples derived from F-scores for PCG-, as well as radar-based heart sound detection showed that both changes in combination are highly significant (*P* < 0.001). An exemplary result of the segmentation algorithm applied on the heart sound signal extracted from the radar data is illustrated in Fig. 8(b).

### Morphology analysis

The morphology of the detected heart sounds is analysed by a cross-correlation-based approach. First, the heart sounds in the PCG and radar data are segmented as described by Springer *et al*.^49^. A reference labelling is used, which employs the ECG R-peaks and T-wave ends as reference points. Only those heart sound pairs of radar and PCG data are utilised in the further steps, in which the heart sounds are detected correctly in both signals, as well as each with a sufficient SNR. Next, since PCG measures acceleration, while radar measures distance, the PCG data is integrated twice to accomplish equal measurands. Due to the different ROIs for radar and PCG, the heart sounds occur with different delays, which is explained in the paragraph “timing analysis”. These delays are estimated and extinguished by cross-correlating both heart sound signals, here named *x*_1_[*n*] and *x*_2_[*n*] with *N* as the larger signal length,

6 $${\hat{R}}_{xy}[m]=\sum _{n=-N}^{N}{x}_{1}^{\ast }[n]\cdot {x}_{2}[n+m],$$

and subsequently searching for the lag with maximum correlation. After shifting one signal by this lag, the lengths of both heart sounds are equalised by removing the non-overlapping parts at both signal ends. The correlation between two equalised heart sounds is finally determined by the Pearson correlation coefficient, at which both signals are normalised so that the autocorrelations at zero lag equals one:

7 $${\hat{R}}_{xy,{\rm{norm}}}[m]=\frac{1}{\sqrt{{\hat{R}}_{xx}\mathrm{[0]}\cdot {\hat{R}}_{yy}\mathrm{[0]}}}\cdot {\hat{R}}_{xy}[m\mathrm{].}$$

### Measurement protocol

**Figure 9:**
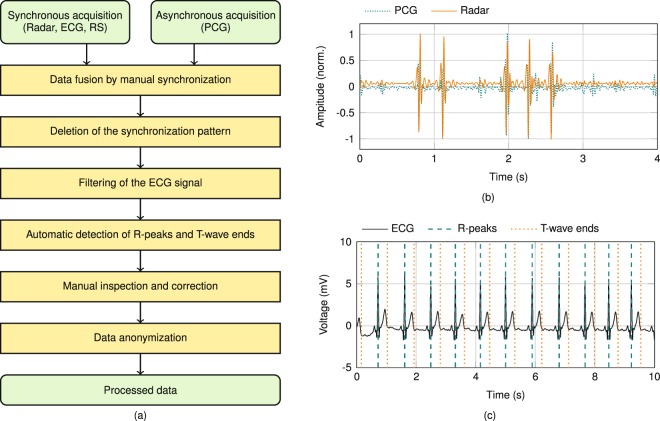
(**a**) Flowchart of the data pre-processing after acquisition. (**b**) Specific pattern caused by tapping in radar and PCG signal to synchronise the asynchronous PCG data to the synchronously acquired radar, ECG and respiration signals. (**c**) Filtered ECG signal with reference labels for detected R-peaks, as well as T-wave ends.

A graphical user interface was implemented in *MATLAB* to establish a communication between PC and BB back end, as well as to support the acquisition and storage of the measurement data. Shortly after starting the data acquisition, the investigator slightly taps several times on the shoulder of the PUT to enable synchronisation between the sensor data, as this tapping is detectable by PCG, as well as by radar. A flowchart of the data pre-processing after their acquisition is shown in Fig. 9(a). As first step, the specific signal caused by tapping is utilised for synchronising the asynchronously acquired PCG data by shifting them along the radar data until overlap of the tapping sequence is achieved, as depicted in Fig. 9(b). Afterwards, initially acquired data including tapping signal are deleted. All three ECG leads are bandpass filtered by using a fourth order Butterworth filter with a passband of 0.5…20.0 Hz. The Pan-Tompkins algorithm^66^ is applied to the filtered ECG data for automatic detection of the QRS complexes, since its R-peak is a crucial parameter to deduce S1 occurrence. Additionally, the endings of the T-waves are localised, in order to infer occurrence of S2 by using the algorithm presented by Zhang *et al*.^67^. Automated extraction of R-peak and ending of the T-wave is checked by the investigator. An exemplary filtered ECG signal with reference labels is depicted in Fig. 9(c). Finally, the dataset consisting of all raw measurement signals plus the information about the measurement scenario is anonymised and stored in a database.

### Subjects and experimental protocol

Measurements were performed with eleven PUT whose anonymised information is presented in Table 2. PUT comprise seven male and four female participants, aged between 24 and 69 years (mean: 34.7 years, median: 26 years) with different body mass indexes (BMI: 23.2 ± 3.6). All PUT were recorded under standardised conditions, i.e. fully clothed, seated comfortably in an arm chair and breathing normally at leisure. In all measurements, the distance between antenna and ROI was set to approximately 20 cm. Additionally, predefined interventions were carried out, comprising changing position (standing or lying down), changing breathing pattern (holding breath for 15 seconds), breathing forcibly fast or slow (20 breaths per minute, and 5 respectively), and after physical exertion (20 squats). Data acquisition was varied following the study protocol over different ROIs, i.e. CL, CR, xiphoid process, 2 L, 4 L, 2 R, 4 R, and back, which are depicted in Fig. 8(a). In total, the database contains 9930 seconds of data, comprising synchronised raw data of ECG, PCG, respiratory sensor, and data derived from radar.

**Table 2:** Overview of PUT characteristics.

PUT^1^	Age	Sex^2^	Height (m)	Weight (kg)	BMI^3^
1	24	M	1,89	100	28,0
2	26	M	1,83	77	23,0
3	26	M	1,82	58	17,5
4	52	M	1,83	86	25,7
5	55	M	1,76	78	25,2
6	25	F	1,67	54	19,4
7	26	M	1,80	76	23,5
8	69	F	1,67	78	28,0
9	24	F	1,73	60	20,0
10	27	F	1,63	52	19,6
11	28	M	1,78	80	25,2

^1^Person-under-test.

^2^M: male, F: female.

^3^Body mass index.

### Data availability

The data that support the findings of this study are available from the corresponding authors upon reasonable request.

### Human subjects

The study was approved by the ethics committee of the Friedrich-Alexander-Universität Erlangen-Nürnberg (No. 85_15B). All research was performed in accordance with relevant guidelines and regulations. The informed consent was obtained from all subjects in human trials.
